# Development and validation of a prediction model for psychological distress in patients with differentiated thyroid cancer undergoing ¹³¹I therapy

**DOI:** 10.3389/fendo.2026.1877295

**Published:** 2026-07-01

**Authors:** Lingling Zeng, Yiming Ma, Suyun Yang

**Affiliations:** 1 Department of Critical Care Medicine, Chongqing University Three Gorges Hospital, Wanzhou, Chongqing, China; 2 College of Nursing, Shanxi Medical University, Taiyuan, Shanxi, China; 3 Department of Nuclear Medicine, First Hospital of Shanxi Medical University, Taiyuan, Shanxi, China; 4 Collaborative Innovation Center for Molecular Imaging of Precision Medicine, Taiyuan, Shanxi, China

**Keywords:** ^131^I therapy, differentiated thyroid cancer, prediction model, psychological distress, risk factors

## Abstract

**Objective:**

To develop and validate a risk prediction model for psychological distress in patients with differentiated thyroid cancer (DTC) undergoing ¹³¹I therapy.

**Methods:**

A total of 247 DTC patients who received ¹³¹I therapy at a tertiary Grade A hospital in Taiyuan between May and December 2025 were enrolled. Independent risk factors for psychological distress were identified to construct the model. Model performance was evaluated using receiver operating characteristic (ROC) curves, the Hosmer-Lemeshow test, and calibration curves.

**Results:**

Multivariate logistic regression identified poor sleep quality, thyroid-stimulating hormone (TSH) stimulation, pulmonary metastasis, social support, radiation exposure concerns, and lower serum 25-hydroxyvitamin D levels as independent risk factors (all *P* < 0.05). The nomogram model achieved an area under the ROC curve (AUC) of 0.835, with a sensitivity of 83.1% and a specificity of 68.2%. The Hosmer-Lemeshow test yielded a χ² of 12.064 (*P* = 0.148). Calibration curves and decision curve analysis (DCA) confirmed good calibration and clinical utility. Internal validation via bootstrap resampling and 10-fold cross-validation produced AUCs of 0.82 and 0.77, respectively.

**Conclusion:**

This internally validated model is a promising tool for predicting psychological distress in DTC patients undergoing ¹³¹I therapy. However, external validation in larger, multicenter cohorts is necessary before routine clinical use can be recommended.

## 1 Introduction

Differentiated thyroid cancer (DTC) is the most common malignancy of the endocrine system, accounting for over 90% of all thyroid cancers (1, 2). Although the prognosis is generally favourable, most patients receive radioiodine (¹³¹I) therapy after thyroidectomy to ablate residual thyroid tissue and potential microscopic metastases, followed by lifelong thyroid hormone replacement therapy (THRT) and long-term surveillance (3–5).

Unlike many aggressive malignancies, DTC carries a long life expectancy, often spanning decades (5). This extended survivorship shifts the clinical focus from oncological outcomes alone to also include patients’ psychological well-being. ¹³¹I therapy, while an essential component of adjuvant treatment, introduces a distinct set of psychosocial stressors. Patients are required to stay in dedicated isolation rooms for several days, a situation that commonly triggers feelings of loneliness, helplessness, and anxiety (6). Concerns about radiation exposure to themselves and their family members, persistent uncertainty over disease recurrence, the burden of lifelong medication, and repeated follow-up visits collectively create a substantial and enduring psychological burden (6, 7).

The high prevalence of anxiety and depression in this population has been well documented. Reported rates range from 38.7% to 52.5% among DTC patients receiving ¹³¹I therapy (8), paradoxically exceeding those observed in patients with breast or colorectal cancer, which have less favourable prognoses (9). Importantly, such emotional distress not only impairs quality of life but may also reduce treatment adherence and indirectly compromise long-term outcomes (10).

Despite the clinical relevance of this issue, the management of psychological distress in DTC patients remains underdeveloped. Most existing studies have focused on describing the prevalence of anxiety and depression or identifying isolated risk factors such as age, sex, or socioeconomic status. A tool that integrates multiple dimensions of risk into an individualized prediction is still lacking. In routine clinical practice, healthcare providers have no reliable means to identify, before treatment, which patients are most likely to develop significant psychological distress, and therefore cannot offer targeted early interventions. Prediction models have been widely used in oncology for prognosis and complication risk stratification, yet no such model has been developed specifically for psychological distress in DTC patients undergoing ¹³¹I therapy.

To address this gap, we aimed to identify independent risk factors for psychological distress in this population and to develop and internally validate a clinically useful nomogram. Such a tool would enable early recognition of high-risk individuals, support decision-making by nurses and physicians, and ultimately improve mental health outcomes and quality of life in DTC patients receiving ¹³¹I therapy.

## 2 Materials and methods

### 2.1 Study participants

Convenience sampling was adopted to recruit patients diagnosed with DTC who underwent ¹³¹I therapy at a tertiary referral hospital in Taiyuan. The recruitment period spanned from May to December 2025.

Inclusion criteria were (1): histopathologically or cytologically confirmed DTC with receipt of ¹³¹I therapy after thyroidectomy (2); age ≥ 18 years (3); cognitively intact and able to communicate verbally.

Exclusion criteria were (1): pre-existing diagnosis of depression, cognitive impairment, or other psychiatric disorders (2); inability to understand or independently complete the questionnaire (3); refusal to participate.

Sample size was calculated based on the events per variable (EPV) criterion, requiring a minimum of 10 outcome events for each candidate predictor. Based on previous literature, we estimated that the final prediction model would include no more than 10 candidate variables. Prior studies reported that the prevalence of anxiety and depression among DTC patients receiving ¹³¹I therapy ranges from 38.7% to 52.5%, with a mean prevalence of 45.6% (8). After accounting for a 5% attrition rate, the minimum required sample size was 230 participants. This study was approved by the Institutional Ethics Committee (approval No. KYLL-2025-263). All enrolled participants provided written informed consent prior to enrolment.

### 2.2 Measurement tools

#### 2.2.1 General information survey form

This questionnaire was developed by the research team to collect demographic, clinical, and laboratory data. Demographic variables included age, gender, marital status, education, working status, family per capita income, medical payment method, height, weight, comorbidities, and ¹³¹I therapy understanding. Clinical characteristics comprised disease duration, number of ¹³¹I therapy sessions, TSH stimulation method (thyroid hormone withdrawal [THW] or recombinant human TSH [rhTSH]), and the presence of tetany or hoarseness, as well as concerns about neck scar, radiation exposure, isolation, and THRT. Laboratory parameters included TSH, serum 25-hydroxyvitamin D [25(OH)D], white blood cell count [WBC], neutrophil count [NEUT], lymphocyte count [LYM], monocyte count [MONO], total cholesterol [TC], high-density lipoprotein cholesterol [HDL-C], and low-density lipoprotein cholesterol [LDL-C].

Comorbidities were identified based on patient self-report and cross-verified with electronic medical records when available; specific conditions (e.g., hypertension, diabetes) were recorded as yes or no. For psychological concerns, the survey included four single-item binary questions (yes/no):

Neck scar concerns: “Are you worried about the neck scar?”

Radiation exposure concerns: “Are you worried about the potential harm of radiation exposure to yourself or your family members during or after ¹³¹I therapy?”

Isolation concerns: “Are you worried about the psychological or practical difficulties of being isolated during ¹³¹I therapy?”

Concerns about THRT: “Are you worried about the need for lifelong thyroid hormone replacement therapy?”

These single-item questions were developed by the research team based on clinical experience and a review of the literature, as no validated scale for these specific concerns was available at the time of study design.

#### 2.2.2 Hospital anxiety and depression scale (HADS)

The HADS was developed by Zigmond and Snaith (11) in 1983 and is widely used to screen for anxiety and depressive symptoms among general hospital inpatients. The scale consists of two subscales: anxiety (HADS-A) and depression (HADS-D), each containing seven items. All items are scored on a 4-point Likert scale (0–3 per item). A score of ≥8 on either subscale was considered to indicate clinically significant anxiety or depressive symptoms. For the purpose of this study, we defined psychological distress as a binary composite outcome: presence of HADS-A ≥8 or HADS-D ≥8. The Cronbach’s α was 0.879 for the total scale and 0.806 for both subscales, confirming satisfactory internal consistency (12).

#### 2.2.3 Social support rating scale (SSRS)

The SSRS, developed by Xiao (13) and adapted to the Chinese cultural context, comprises 10 items across three dimensions: objective social support, subjective social support, and utilization of social support. Total scores range from 12 to 66, with ≤22 indicating low, 23–44 moderate, and 45–66 high social support. The scale has been widely used in Chinese populations and has demonstrated satisfactory reliability and validity.

#### 2.2.4 Pittsburgh sleep quality index (PSQI)

PSQI was devised by Buysse et al. (14) in 1989. This study adopted the Chinese version translated and revised by Liu et al. (15). It consists of 18 items categorized into seven components: sleep quality, sleep latency, sleep efficiency, sleep duration, sleep disturbances, daytime dysfunction, and use of hypnotic medications. Each component is scored from 0 to 3, and the total score ranges from 0 to 21. A cut-off of ≥8 was used to define poor sleep quality, based on the Chinese validation study (15). The Cronbach’s α coefficients for the individual items and the seven components were 0.845 and 0.832, respectively (16).

### 2.3 Data collection and quality control

To ensure data quality and minimize response bias, we implemented the following procedures. A dedicated research team was assembled, and all members were trained to a standardized protocol. Clear definitions of the study objectives, content, data-collection procedures, and quality-control criteria were established to maintain consistency throughout the study.

Eligible patients and their family members received a detailed explanation of the study purpose, procedures, and questionnaire requirements. Written informed consent was obtained only after participants fully understood the study protocol and voluntarily agreed to participate.

Before ¹³¹I administration, trained investigators conducted on-site surveys and guided patients to complete the SSRS, PSQI, and HADS via a quick-response (QR) code. All psychological assessments were therefore completed at this pre-treatment time point; thus, the model predicts psychological distress specifically at this assessment point rather than during or after ¹³¹I therapy. For individuals who could not complete the questionnaires independently due to low education level or visual impairment, researchers offered item-by-item interpretation and assisted with form completion. All questionnaires were reviewed immediately upon completion and collected only after verification of completeness.

Sociodemographic characteristics, disease diagnosis and treatment records, and laboratory test results were extracted from the electronic medical record system the following day. After overall data collection, two researchers independently entered all data into the study database and cross-checked the data to ensure integrity and accuracy.

To enhance the clinical validity of the psychological outcome, a licensed psychiatrist was consulted to provide an independent review of the study design, including the selection of the Hospital Anxiety and Depression Scale (HADS), the definition of the composite outcome (psychological distress), and the choice of relevant variables.

### 2.4 Statistical analysis

All statistical analyses were conducted using SPSS 25.0 and R 4.5.1. Normally distributed continuous variables were expressed as mean ± standard deviation (SD), and between-group differences were compared using the independent samples t-test. Non-normally distributed continuous variables were presented as median (interquartile range, IQR), with intergroup comparisons performed using the Mann-Whitney U test. Categorical variables were described as frequencies and percentages (n, %), and group differences were examined using the χ² test or Fisher’s exact test.

Variables with *P* < 0.10 in univariate analysis were included for collinearity assessment. Variables with a variance inflation factor (VIF) ≥10 or tolerance <0.1 were excluded from subsequent modeling. Backward stepwise logistic regression based on the likelihood ratio test was employed to screen independent risk factors and establish the prediction model. A nomogram was constructed for intuitive model visualization.

Model validation was conducted via bootstrap resampling with 1,000 iterations and 10-fold cross-validation. The receiver operating characteristic (ROC) curve, area under the curve (AUC), sensitivity, specificity, and Hosmer-Lemeshow test were used to evaluate predictive performance. Calibration curves and decision curve analysis (DCA) were plotted to assess calibration and clinical utility. A *P* value <0.05 was considered statistically significant.

## 3 Results

### 3.1 Univariate analysis of psychological distress in DTC patients undergoing ¹³¹I therapy

A total of 247 patients with DTC receiving ¹³¹I therapy were enrolled, including 88 males and 159 females. Of these, 77 patients (31.17%) met the criteria for psychological distress (i.e., HADS-A ≥ 8 and/or HADS-D ≥ 8) and were assigned to the distress group, while the remaining 170 (68.83%) were assigned to the non-distressed group.

Variables showing between-group differences at the threshold of *P* < 0.10 in univariate analysis included: gender, medical payment method, understanding of ¹³¹I therapy, poor sleep quality, social support level, TSH stimulation method, pulmonary metastasis, neck scar concerns, radiation exposure concerns, concerns about THRT, and serum 25(OH)D level (**Supplementary Table 1**). Complete TNM staging was not available for all patients due to variability in surgical records from referring hospitals; therefore, only the presence of pulmonary metastasis was included as a marker of advanced disease.

### 3.2 Multivariate logistic analysis of psychological distress

Collinearity testing was performed on the 11 variables that yielded *P* < 0.10 in univariate analysis. No multicollinearity was detected (all VIF <5; **Table 1**). The occurrence of psychological distress was set as the dependent variable. All variables that were statistically significant in univariate analysis—gender, medical payment method, understanding of ¹³¹I therapy, poor sleep quality, social support level, TSH stimulation method, pulmonary metastasis, neck scar concerns, radiation exposure concerns, concerns about THRT, and serum 25(OH)D level—were entered as independent variables. Backward stepwise logistic regression using the likelihood ratio method was then performed. The coding scheme for independent variables is shown in **Table 2**.

**Table 1:** Multicollinearity analysis.

Variable	Tolerance	VIF
Gender	0.826	1.211
Medical payment methods	0.897	1.114
^131^I therapy understanding	0.928	1.078
Poor sleep quality	0.873	1.145
Social support level	0.946	1.057
TSH stimulation	0.915	1.093
Pulmonary metastasis	0.942	1.062
Neck scar concerns	0.803	1.245
Radiation exposure concerns	0.858	1.166
Concerns about THRT	0.903	1.107
25(OH)D	0.960	1.042

VIF, Variance Inflation Factor.

**Table 2:** Coding of independent variables.

Variable	Variable coding
Gender	Male=1, Female=2
Medical payment methods	Provincial/municipal medical insurance = 1, Urban and rural residents medical insurance = 2, Self-paid = 3
^131^I therapy understanding	Unclear = 1, Moderately clear = 2, Clear = 3
Poor sleep quality	No = 0, Yes = 1
Social support level	Low = 1, Moderate = 2, High = 3
TSH stimulation	THW=1, rhTSH=2
Pulmonary metastasis	No = 0, Yes = 1
Neck scar concerns	No = 0, Yes = 1
Radiation exposure concerns	No = 0, Yes = 1
Concerns about THRT	No = 0, Yes = 1
25(OH)D	Original value

Multivariate logistic regression identified poor sleep quality, TSH stimulation method (THW vs. rhTSH), pulmonary metastasis, social support level, radiation exposure concerns, and serum 25(OH)D level as independent factors associated with psychological distress (all *P* < 0.05; **Table 3**). Notably, the odds ratio for pulmonary metastasis was high (16.49) with a wide 95% confidence interval (3.25–83.74), indicating statistical imprecision due to the small subgroup size (n = 14, 5.7% of the cohort). Nevertheless, the association remained significant (*P* < 0.001), suggesting a clinically meaningful effect that warrants further validation.

**Table 3:** Multivariable logistic regression analysis of psychological distress in patients with DTC undergoing ¹³¹I therapy.

Variable	B	SE	Wald	*P*	*OR*	95%*CI*
Poor sleep quality (No)						
Yes	0.808	0.352	5.268	0.022	2.244	(1.125, 4.476)
Social support (Low)						
Moderate	-0.979	0.407	5.793	0.016	0.376	(0.169, 0.834)
High	-1.508	0.559	7.271	0.007	0.221	(0.074, 0.662)
TSH stimulation(THW)						
rhTSH	-0.929	0.451	4.242	0.039	0.395	(0.163, 0.956)
Pulmonary metastasis (No)						
Yes	2.803	0.829	11.431	<0.001	16.491	(3.248, 83.735)
Radiation exposure concerns(No)						
Yes	1.353	0.359	14.215	<0.001	3.869	(1.915, 7.817)
25(OH)D	-0.032	0.010	10.168	0.001	0.968	(0.949,0.988)
Constant	0.574	0.850	0.457	0.499	1.776	–

B: regression coefficient; SE: standard error; Wald: Wald chi-square test; OR: odds ratio.

The final logistic regression equation was:

Logit (*P*) = 0.574 + 0.808× (poor sleep quality) − 0.979 × (moderate social support) − 1.508 × (high social support) − 0.929 × (rhTSH stimulation) + 2.803 × (pulmonary metastasis) + 1.353 × (radiation exposure concerns) − 0.032 × 25(OH)D level.

### 3.3 Development and validation of a nomogram model

**Figure 1:**
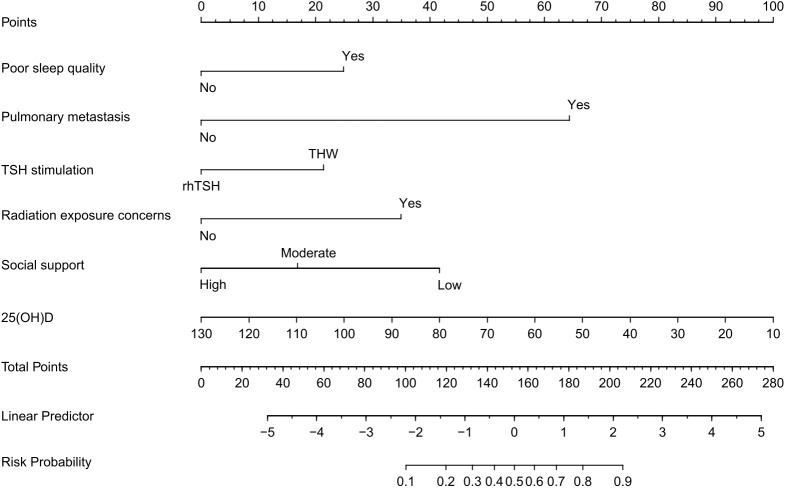
Nomogram for predicting psychological distress in DTC patients undergoing ¹³¹I therapy.

Using the six independent determinants identified by logistic regression, a nomogram predictive model for psychological distress was established using the *rms* package in R 4.5.1 (**Figure 1**). The nomogram allows assignment of individual points to each predictor: poor sleep quality, TSH stimulation method, pulmonary metastasis, social support level, radiation exposure concerns, and serum 25(OH)D level. Summation of these points yields a total score, from which the corresponding predicted probability reflects an individual’s likelihood of developing psychological distress.

Internal validation was performed using 1,000 bootstrap resampling iterations, yielding an AUC of 0.82 (95% CI: 0.772–0.872). Ten-fold cross-validation with 200 iterative runs returned an AUC of 0.77 (95% CI: 0.534–0.971).

### 3.4 Evaluation of the the prediction model

**Figure 2:**
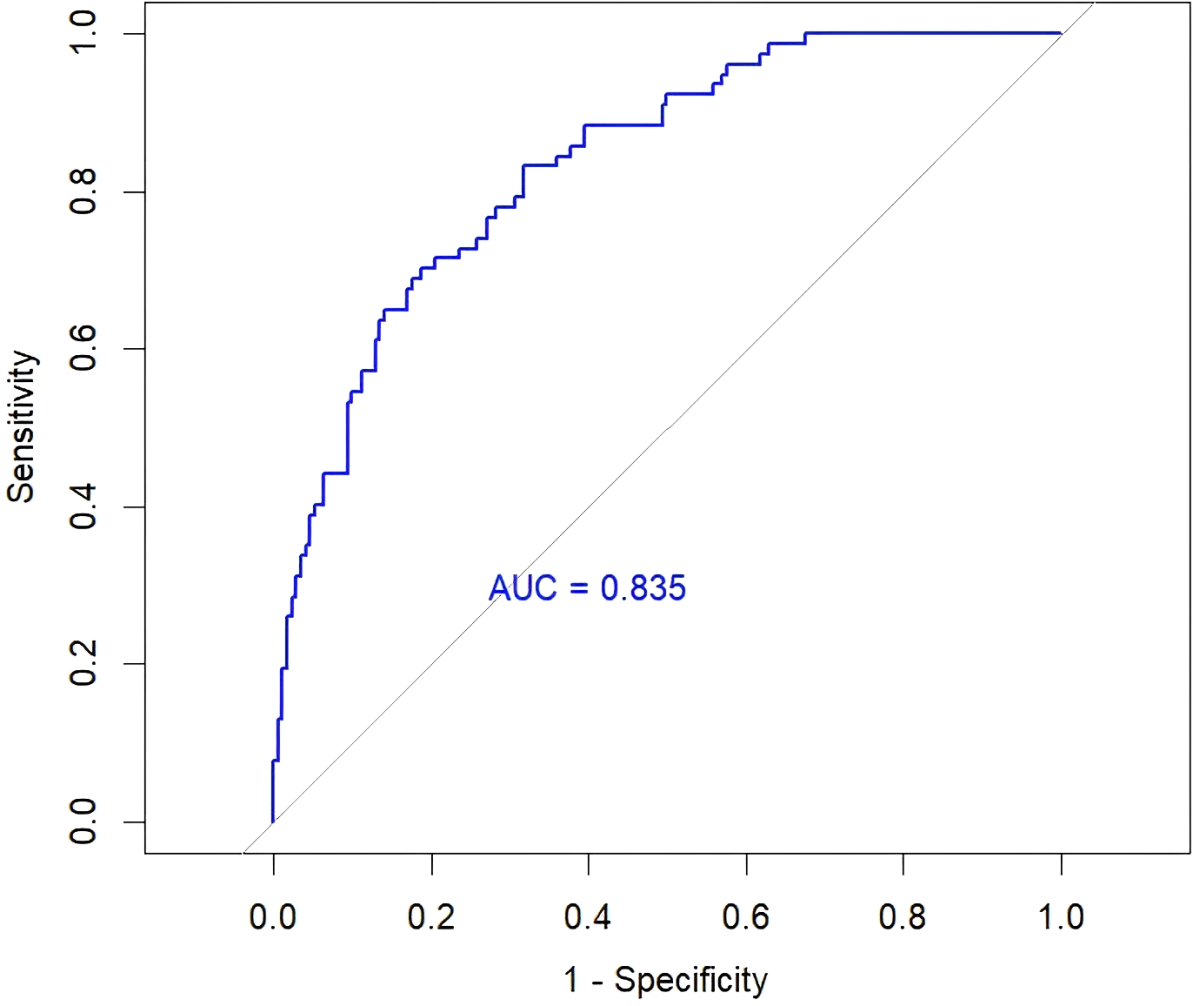
ROC curve of the prediction model. ROC, Receiver operating characteristic; AUC, the area under the ROC.

ROC curve analysis produced an AUC of 0.835 (95% CI: 0.784–0.887). The maximum Youden index was 0.514, with an optimal cut-off value of 0.256. At this threshold, the model exhibited a sensitivity of 83.1% and a specificity of 68.2% (**Figure 2**).

**Figure 3:**
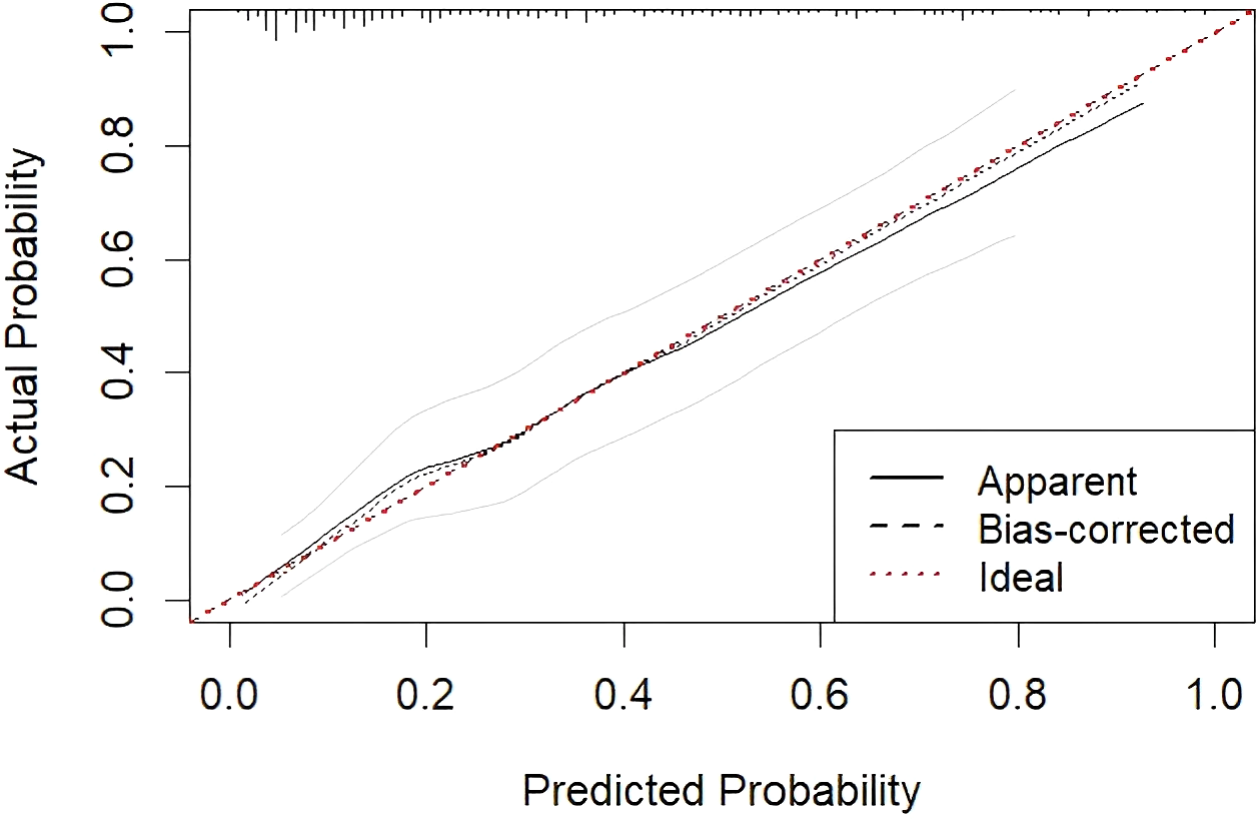
Calibration curve of the prediction model.

The Hosmer-Lemeshow test yielded a χ² value of 12.064 (*P* = 0.148), indicating no significant discrepancy between predicted and observed outcomes and demonstrating good model fit. The calibration curve (**Figure 3**) closely followed the ideal diagonal line, with a mean absolute error of 0.017, confirming satisfactory calibration of the nomogram.

**Figure 4:**
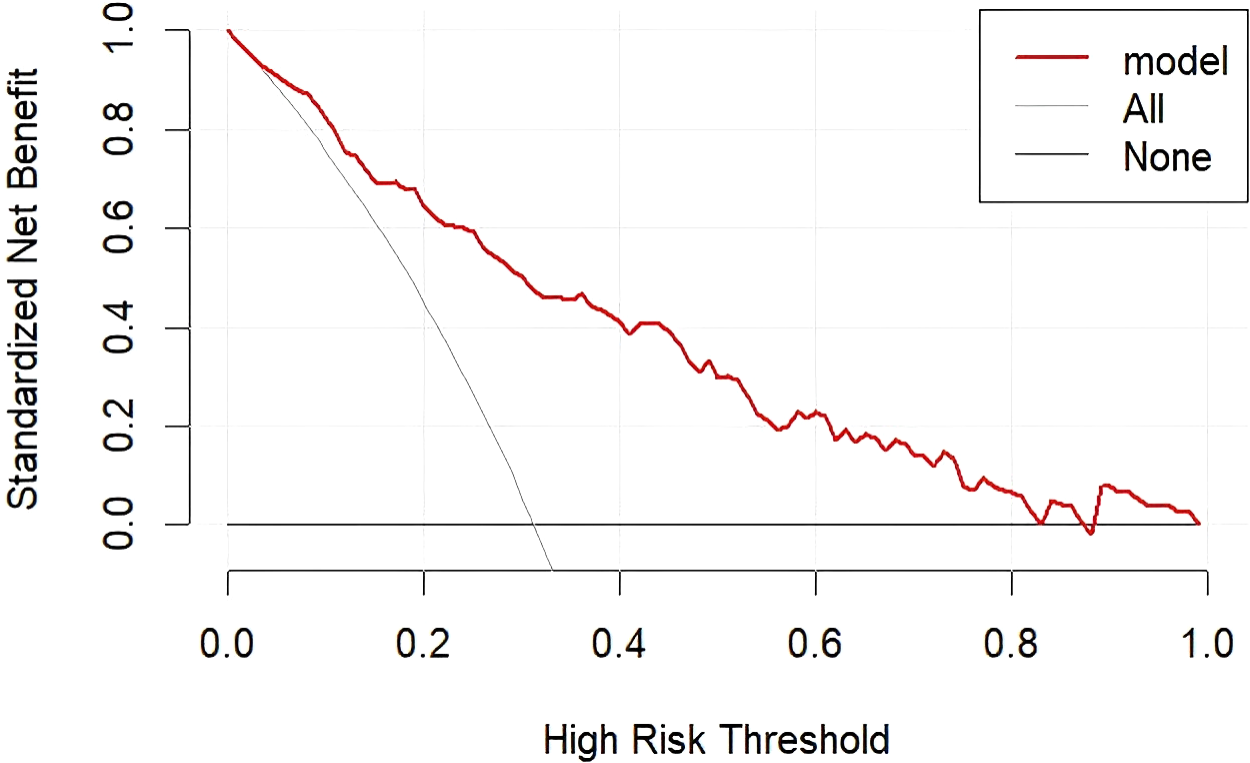
DCA of the prediction model. DCA, Decision curve analysis.

Decision curve analysis (**Figure 4**) showed that within a wide range of high-risk thresholds (5%–85%), the red predictive curve remained consistently above the horizontal black line (strategy of no intervention for all) and the grey oblique line (strategy of universal intervention). In this derivation cohort, the decision curve suggests that the model offers net benefit over treat-all or treat-none strategies across a range of threshold probabilities, supporting its potential utility as a decision aid. Confirmation in external validation cohorts is an important next step.

## 4 Discussion

In this study, the pooled prevalence of psychological distress among DTC patients undergoing ¹³¹I therapy was 31.17%, which is relatively lower than rates reported in previous domestic and international studies of similar populations. Qiao et al. examined 112 patients with thyroid cancer and reported psychological distress prevalence rates of 41.08% and 33.93%, respectively. In a clinical survey of 80 DTC patients conducted by Qin et al. (8), the prevalence of anxiety reached 38.75%, while that of depression was as high as 52.5%. Another prospective study enrolling 200 participants further confirmed that approximately 36% of patients had already developed anxiety or depression prior to ¹³¹I therapy (17).

Several factors may account for these discrepancies. First, there is notable heterogeneity across study populations, including variations in age distribution, disease course, treatment regimens, and social support status, all of which can directly affect patients’ psychological well-being. Second, different assessment tools were used across studies. Differences in scale dimensions and scoring criteria may yield divergent results for similar patient cohorts, thereby undermining comparability of prevalence estimates. Third, inherent differences in study design—such as sample size and the timing of data collection (e.g., pre-treatment, peri-treatment, or follow-up)—may also confound the findings. Despite these numerical inconsistencies, accumulating evidence consistently suggests that anxiety and depression are highly prevalent psychological comorbidities throughout the course of ¹³¹I therapy for thyroid cancer, and such emotional disturbances substantially impair patients’ health-related quality of life (17–19). Therefore, clinical practitioners should pay sufficient attention to the psychological status of this patient group. Routine psychological assessment integrated into standard nursing and management protocols for DTC patients undergoing ¹³¹I therapy is recommended. Early identification of at-risk individuals coupled with targeted interventions can effectively alleviate emotional distress and optimize patients’ overall quality of life.

Poor sleep quality was significantly associated with psychological distress (*OR* = 2.244), indicating it may act as a notable correlate. This finding is consistent with previous studies. For example, Hong et al. (20) noted that poor sleep quality often coexists with anxious and depressive symptoms. A meta-analysis further identified insomnia as a prominent predictor of anxiety and depression (21). Epidemiological evidence has revealed that over 90% of patients with major depressive disorder have concurrent sleep disturbance (22), and the severity of anxiety symptoms tends to correlate with deteriorated sleep function (23). Mechanistically, the neuroplasticity hypothesis partially illustrates the linkage between sleep quality and depression. This hypothesis proposes that impaired synaptic plasticity is a core pathological feature of depression. Meanwhile, insufficient sleep may be linked to heightened vulnerability to neuroplastic dysfunction, a state that could potentially correlate with an increased likelihood of developing depression (21). Additionally, sleep, especially rapid eye movement (REM) sleep, undertakes a critical role in emotional regulation. It mitigates negative emotional intensity and attenuates physiological and neural reactions to adverse stimuli. In contrast, sleep deprivation may interfere with the generalization of fear extinction memory and could hinder emotional recovery (24).

It is worth noting that the optimal PSQI cut-off varies across populations. A recent systematic review and meta-analysis by Mollayeva et al. concluded that the commonly used threshold of >5 is not universally applicable, as the presentation of sleep disturbances differs substantially between clinical and non-clinical samples (25). In oncology populations, factors such as disease-related fear, psychological sequelae of a cancer diagnosis, and treatment-specific side effects often elevate background sleep complaints, thereby necessitating a higher threshold to accurately identify clinically significant sleep disturbance (26, 27). Accordingly, Carpenter and Andrykowski provided early evidence specifically recommending against the original >5 cut-off in cancer patients, proposing an elevated threshold of >8 (28). This recommendation has since been validated cross-culturally; for instance, Kotronoulas et al. demonstrated robust psychometric properties of the Greek version of the PSQI using a cut-off of ≥8 in patients receiving chemotherapy, supporting its validity in oncology settings (29). In alignment with this international evidence and the Chinese validation study by Liu et al., which recommended a cut-off of >7 (i.e., ≥8) for the general Chinese population (30), our study adopted a cut-off of ≥8. By applying this stringent, cancer-specific criterion, we aimed to minimize exposure misclassification and obtain a more precise estimate of the association between sleep disturbance and psychological distress.

Social support refers to the material, emotional, and informational assistance that individuals obtain through social interactions, serving as a critical resource for coping with stress and preserving physical and mental well-being (31). The present study identified social support level as a key factor influencing anxiety and depression in DTC patients receiving ¹³¹I therapy, with higher social support linked to a lower risk of developing anxious and depressive symptoms. This finding aligns well with previous investigations (32–34). Adequate social support has been associated with a strengthened sense of belonging and self-confidence, as well as greater feelings of being valued, which may buffer the adverse impacts of negative life events on physical and mental health (35). Additionally, social support facilitates patients’ post-treatment adaptation and exerts positive modulatory effects on physiological mechanisms associated with disease recovery (36). Such benefits may be attributed to the favourable influence of social support on health-related behaviours. For instance, sustained social support can bolster patients’ confidence in managing treatment side effects and improve their medication adherence (37). Given that isolation following radioiodine administration may exacerbate feelings of loneliness, clinical practice should prioritize strategies to enhance patients’ social support. Measures such as encouraging family companionship and establishing peer support platforms can effectively elevate social support levels, thereby reducing the likelihood of anxiety and depression in this patient population.

TSH stimulation method was another independent factor. This study demonstrated that compared with THW, ¹³¹I therapy assisted by rhTSH was associated with a lower risk of anxiety and depression in DTC patients. Prior studies have also documented that patients undergoing THW exhibit more severe negative emotions than those in the rhTSH group, adversely affecting their quality of life (38). Chow et al. (39) likewise indicated that THW-induced hypothyroidism before ¹³¹I therapy is a major contributor to deteriorated mental health in these patients. Two potential mechanisms may underlie this phenomenon. On the one hand, THW can induce clinically significant hypothyroidism (40). Hypothyroidism is commonly accompanied by neuropsychiatric symptoms such as depression, delusions, and hallucinations, as well as somatic complaints including fatigue, weight gain, and muscle spasms; all these manifestations markedly impair patients’ quality of life (41, 42). On the other hand, rhTSH application shortens the waiting interval between surgery and subsequent ¹³¹I therapy (43). Thyroid cancer patients generally have a favourable prognosis and prolonged survival. Accordingly, maintaining quality of life has become a pivotal goal in disease management. In clinical decision-making, clinicians should comprehensively consider patients’ comorbidities, psychological status, and economic conditions, and fully inform them of the benefits and drawbacks of THW and rhTSH preparation regimens. This is especially important for patients with a history of emotional disorders or those who pursue a high quality of life.

Pulmonary metastasis is an independent risk factor for the development of anxiety and depression in DTC patients receiving ¹³¹I therapy. He et al. (44) similarly demonstrated that thyroid cancer patients with metastatic lesions endure greater psychological distress and health-related worries. Pulmonary metastasis is generally linked to poorer prognosis, which may serve as a key contributor to exacerbated emotional distress in affected patients (45). As the disease advances to an advanced stage, worsening clinical symptoms, more frequent treatment cycles, and declining prognostic expectations collectively lead to a substantial elevation in patients’ psychological burden (46). Bruno et al. further demonstrated via structural equation modelling that disease stage moderates the association between perceived social support and psychological distress. The buffering effect of social support against anxiety and depression tends to be attenuated in individuals with advanced cancer (47).

Radiation exposure concerns were identified as a critical risk factor. ¹³¹I therapy selectively ablates residual thyroid tissue and metastatic lesions through targeted radiation effects, which inevitably exposes surrounding normal tissues to a certain level of radiation during treatment (48). For a period after intervention, patients continuously emit trace amounts of radiation via bodily fluids such as saliva and urine, placing cohabiting family members at potential risk of low-dose radiation exposure (49). These underlying radiation-related risks commonly evoke persistent apprehension and concern among patients. Our study found that radiation exposure worries were a critical risk factor for anxiety and depression. Su et al. (50) also demonstrated that patient apprehension about radiation-related issues easily triggers panic, anxiety, and other negative emotional disturbances. Such worries may even impair treatment adherence and compromise overall therapeutic outcomes, a scenario largely attributable to patients’ inadequate understanding of ¹³¹I treatment fundamentals. The International Atomic Energy Agency (IAEA) stipulates an annual radiation dose limit of 1 mSv for the general population, while the National Council on Radiation Protection and Measurements (NCRP) allows a more lenient exposure threshold of up to 5 mSv for non-pregnant adults caring for family recipients of radionuclide therapy (49). Standardised radiation safety education should now become an integral component of routine clinical care. Clinicians should, throughout the full treatment trajectory—especially for patients with overt pre-existing concerns—clarify therapeutic principles, actual radiation dosage levels, protective protocols, and relevant safety evidence using visual materials and standardised guidelines.

Serum 25(OH)D levels were inversely associated with psychological distress. Following total thyroidectomy, a subset of patients develop hypoparathyroidism, which subsequently progresses to hypocalcaemia (51). Our study found that lower serum 25(OH)D levels were associated with higher odds of psychological distress, consistent with previous observational studies linking vitamin D to mood disorders (52–54). The underlying mechanisms are complex and may involve neuroinflammation, neurotransmitter modulation, and gut-brain axis regulation (55), but these pathways remain largely speculative and cannot be directly tested in the present cross-sectional design. Notably, our observational data do not establish causality; low 25(OH)D could be a consequence rather than a cause of psychological distress. Moreover, clinical trials of vitamin D supplementation for anxiety and depression have yielded mixed results to date. Therefore, while our findings suggest an association, they do not provide evidence that vitamin D supplementation would reduce psychological distress in DTC patients undergoing ¹³¹I therapy. Future prospective studies, including randomized controlled trials, are needed to clarify whether a causal relationship exists and whether supplementation offers therapeutic benefit.

## Limitations

Several methodological, statistical and design-related limitations must be acknowledged to contextualize the interpretation of our predictive model for psychological distress among differentiated thyroid carcinoma (DTC) patients undergoing radioiodine therapy.

First, with regard to pulmonary metastasis—one of the six independent determinants in our model—two important caveats apply. (a) The wide 95% confidence interval (3.25–83.74) and the high odds ratio (16.49) reflect the small number of affected patients (n = 14, 5.7% of the cohort), leading to statistical imprecision. Although the association remained highly significant (*P* < 0.001), the magnitude of the effect should be interpreted with caution. (b) Comprehensive TNM staging and local invasion status could not be included because a substantial proportion of patients underwent thyroidectomy at outside hospitals, and complete histopathological data were not uniformly available. Pulmonary metastasis was the most reliably documented indicator of advanced disease, but residual confounding by unmeasured oncological variables (e.g., tumour size, extrathyroidal extension, lymph node burden) cannot be ruled out. Clinically, the strong observed association is plausible: a diagnosis of pulmonary metastasis in DTC often signals a poorer prognosis, which may profoundly elevate psychological distress. Future multicenter studies with larger samples—particularly more patients with distant metastasis—and standardised collection of full staging data are needed to validate and refine this finding.

Second, our primary composite endpoint for psychological distress was defined by HADS-Anxiety ≥8 and/or HADS-Depression ≥8. This binary composite outcome does not permit separate quantification or prediction of anxiety and depressive symptomatology as distinct clinical entities. While this composite metric fulfilled the pragmatic objective of identifying any patient experiencing clinically relevant distress in our initial model development, it obscures potentially divergent risk factor profiles for isolated anxiety versus isolated depression. Large, adequately powered subsequent studies should develop discrete prediction models to differentiate predictors of anxiety and depression independently.

Third, this study employed convenience sampling from a single tertiary hospital, which may limit the representativeness of the sample. More importantly, the prediction model was validated only through internal methods, specifically bootstrap resampling and 10-fold cross-validation. With only 77 patients experiencing the outcome event, the risk of overfitting cannot be ignored. While internal validation provides some reassurance against overfitting, it does not substitute for external validation. The wide 95% confidence interval of the cross-validated AUC (0.534-0.971) reflects considerable uncertainty in model performance, likely attributable to the modest sample size and the low prevalence of certain predictors (e.g., pulmonary metastasis). Consequently, the model’s generalisability to broader clinical populations remains to be established. External validation in an independent, multicenter cohort is essential before the model can be recommended for routine clinical use.

Fourth, the cross-sectional nature of this study precludes any causal inference. All data were collected at a single time point, meaning we can only report associations between risk factors and psychological distress without establishing a temporal sequence. Reverse causality cannot be ruled out; for example, poor sleep quality and low perceived social support may be consequences, rather than predictors, of pre-existing emotional distress. Future longitudinal studies with repeated measurements are needed to clarify the direction of these relationships.

Fifth, several established potential confounding variables were not captured within our data collection framework, including alcohol consumption, tobacco smoking history, and pre-existing neurological comorbidities such as stroke or Parkinson’s disease. Omission of these covariates may leave residual confounding unaccounted for in our multivariable regression model. Future predictive research should integrate standardized assessments of lifestyle exposures and neurological medical history to improve model adjustment and predictive accuracy.

Sixth, patient concerns specific to radioiodine therapy—including worries relating to neck scarring, radiation exposure, social isolation, and lifelong thyroid hormone replacement—were measured using custom single-item binary (yes/no) questions developed by our research team via clinical observation and narrative literature review, rather than established psychometric multi-item scales. Although these bespoke items directly query disease- and treatment-specific worries salient to patients receiving ¹³¹I therapy, their binary response format inherently discards granular data on worry severity, failing to capture the graded complexity of patient emotional concerns. Further, without formal psychometric evaluation, the reliability, construct validity and responsiveness of these single-item measures remain uncharacterized, introducing potential measurement bias. Subsequent research should deploy fully validated multi-item patient-reported outcome tools to comprehensively quantify these treatment-specific psychological concerns.

Notwithstanding the aforementioned limitations, this work carries incremental clinical and methodological value. We developed an internally validated multivariable prediction model to identify DTC patients at heightened risk of psychological distress during radioiodine therapy, which may offer a candidate risk stratification tool to address the unmet clinical demand in routine endocrine oncology care. While our findings require cross-cohort replication and external validation prior to broad clinical implementation, this model provides an evidence-based preliminary framework that could support early, targeted psychosocial screening and supportive interventions for vulnerable patient subgroups.

## 5 Conclusion

This study identified poor sleep quality, low social support, TSH stimulation (THW), pulmonary metastasis, concerns about radiation exposure, and lower serum 25(OH)D levels as independent determinants of psychological distress in DTC patients undergoing ¹³¹I therapy. The established prediction model demonstrated good discrimination and calibration in internal validation. It offers a preliminary evidence-based tool for clinicians to identify individuals at high risk for psychological distress within similar clinical settings. However, external validation in independent cohorts is necessary before the model can be recommended for broader clinical use.

## Data Availability

The original contributions presented in the study are included in the article/**Supplementary Material**. Further inquiries can be directed to the corresponding author.
