# Supplementary material for: Development and validation of a prediction model for psychological distress in patients with differentiated thyroid cancer undergoing ¹³¹I therapy
Source: Front Endocrinol (Lausanne). 2026 Jul 1;17:1877295. doi: 10.3389/fendo.2026.1877295 (PMC13368564; doi:10.3389/fendo.2026.1877295)
Supplement: Supplementary file 1 [file Table1.docx]

Supplementary Material

# Supplementary Tables

**Table S1 Univariate analysis of psychological distress in patients with DTC receiving ¹³¹I therapy**

| Variable | Psychological distress group（n=77） | Non-psychological distress group（n=170） | Statistical values | *P* |
| --- | --- | --- | --- | --- |
| Age(years) | 46(39, 55) | 45(34, 56) | -0.970^2)^ | 0.332 |
| Gender |  |  | 14.847^1)^ | <0.001 |
| Male | 14(18.2%) | 74(43.5%) |  |  |
| Female | 63(81.8%) | 96(56.5%) |  |  |
| Marital status |  |  | 0.282^3)^ | 1.000 |
| Married | 73(94.8%) | 158(92.9%) |  |  |
| Single | 3(3.9%) | 8(4.7%) |  |  |
| Divorced/Widowed | 1(1.3%) | 4(2.4%) |  |  |
| Education |  |  | 6.836^1)^ | 0.145 |
| Primary school and below | 8(10.4%) | 17(10%) |  |  |
| Junior high school | 31(40.3%) | 52(30.6%) |  |  |
| Senior high school | 7(11.7%) | 27(15.9%) |  |  |
| College | 16(20.8%) | 24(14.1%) |  |  |
| Bachelor degree and above | 13(16.9%) | 50(29.4%) |  |  |
| Family per capita income |  |  | 4.253^1)^ | 0.119 |
| < 3000 | 35(45.5%) | 63(38.8%) |  |  |
| 3000 ~ 5000 | 34(44.2%) | 68(40%) |  |  |
| > 5000 | 8(10.4%) | 36(21.2%) |  |  |
| Medical payment methods |  |  | 4.486^3)^ | 0.067 |
| Provincial/municipal medical insurance | 32(41.6%) | 90(52.9%) |  |  |
| Urban and rural residents medical insurance | 44(57.1%) | 80(47.1%) |  |  |
| Self-paid | 1(1.3%) | 0(0%) |  |  |
| Working status |  |  | 0.830^1)^ | 0.362 |
| Unemployed / Retired | 50(64.9%) | 100(58.8%) |  |  |
| Employed | 27(35.1%) | 70(41.2%) |  |  |
| ^131^I therapy understanding |  |  | 4.636^1)^ | 0.098 |
| Unclear | 21(27.3%) | 29(17.1%) |  |  |
| Moderately clear | 52(67.5%) | 123(72.4%) |  |  |
| Clear | 4(5.2%) | 18(10.6%) |  |  |
| BMI (kg/m^2^) | 25.25(22.50, 27.91) | 24.46(22.29, 26.64) | -0.906^2)^ | 0.365 |
| Poor sleep quality |  |  | 21.276^1)^ | <0.001 |
| Yes | 49(63.6%) | 55(32.4%) |  |  |
| No | 28(36.4%) | 115(67.6%) |  |  |
| Comorbidities |  |  | 1.888^1)^ | 0.169 |
| Yes | 24(31.2%) | 39(22.9%) |  |  |
| No | 53(68.8%) | 131(77.1%) |  |  |
| Social support |  |  | 12.959^1)^ | 0.002 |
| Low | 32(41.6%) | 34(20%) |  |  |
| Moderate | 35(45.5%) | 99(58.2%) |  |  |
| High | 10(13%) | 37(21.8%) |  |  |
| Disease duration (months) |  |  | 2.954^3)^ | 0.397 |
| ≤ 3 | 65(84.4%) | 142(83.5%) |  |  |
| 3 ~ 6 | 5(6.5%) | 13(7.6%) |  |  |
| 6 ~ 12 | 6(7.8%) | 7(4.1%) |  |  |
| > 12 | 1(1.3%) | 8(4.7%) |  |  |
| Number of ^131^I therapy |  |  | 1.504^3)^ | 0.905 |
| 1 | 67(87%) | 149(87.6%) |  |  |
| 2 | 6(7.8%) | 14(8.2%) |  |  |
| 3 | 2(2.6%) | 4(2.4%) |  |  |
| 4 | 2(2.6%) | 2(1.2%) |  |  |
| 5 | 0(0%) | 1(0.6%) |  |  |
| TSH stimulation |  |  | 9.837^1)^ | 0.002 |
| THW | 66(85.7%) | 113(66.5%) |  |  |
| rhTSH | 11(14.3%) | 57(33.5%) |  |  |
| Hoarseness |  |  | 2.353^1)^ | 0.125 |
| Yes | 13(16.9%) | 17(10%) |  |  |
| No | 64(83.1%) | 153(90%) |  |  |
| Tetany |  |  | -^3)^ | 0.466 |
| Yes | 4(5.2%) | 5(2.9%) |  |  |
| No | 73(94.8%) | 165(97.1%) |  |  |
| Pulmonary metastasis |  |  | -^3)^ | <0.001 |
| Yes | 11(14.3%) | 3(1.8%) |  |  |
| No | 66(85.7%) | 167(98.2%) |  |  |
| Neck scar concerns |  |  | 23.249^1)^ | <0.001 |
| Yes | 54(70.1%) | 63(37.1%) |  |  |
| No | 64(83.1%) | 153(90%) |  |  |
| Radiation exposure concerns |  |  | 24.843^1)^ | <0.001 |
| Yes | 50(64.9%) | 53(31.2%) |  |  |
| No | 27(35.1%) | 117(68.8%) |  |  |
| Isolation concerns |  |  | 0.052^1)^ | 0.820 |
| Yes | 22(28.6%) | 51(30%) |  |  |
| No | 55(71.4%) | 119(70%) |  |  |
| Concerns about THRT |  |  | 3.186^1)^ | 0.074 |
| Yes | 33(42.9%) | 53(31.2%) |  |  |
| No | 44(57.1%) | 117(68.8%) |  |  |
| TSH (mIU/L) | 100(78.56, 100) | 100(85.50, 100) | -0.375^2)^ | 0.707 |
| 25(OH)D (nmol/L) | 46.09(35.04, 61.67) | 53.76(43.33, 69.44) | -3.278^2)^ | <0.001 |
| WBC (* 10^9^/L) | 5.5(4.75, 7.05) | 6(5.1, 7) | -1.276^2)^ | 0.202 |
| NEUT (* 10^9^/L) | 3.74(3, 4.57) | 3.96(3.19, 4.87) | -1.236^2)^ | 0.216 |
| LYM (* 10^9^/L) | 1.55(1.33, 1.98) | 1.68(1.37, 2.04) | -1.155^2)^ | 0.248 |
| MONO (* 10^9^/L) | 0.24(0.17, 0.31) | 0.24(0.19, 0.3) | -0.387^2)^ | 0.699 |
| TC (mmol/L) | 6.55(5.06, 7.61) | 6.34(5.21, 7.50) | -0.551^2)^ | 0.582 |
| HDL-C(mmol/L) | 1.52(1.29, 1.85) | 1.56(1.26, 1.83) | -0.173^2)^ | 0.863 |
| LDL-C (mmol/L) | 4(3, 4.60) | 3.78(3.12, 4.63) | -0.435^2)^ | 0.664 |

1): χ² value；2): *Z* value；3): Fisher exact test
